# In Situ Fabrication and Static Contact Resistance of CdMoO_4_ Reinforced Cu Matrix Composites

**DOI:** 10.3390/ma15207206

**Published:** 2022-10-16

**Authors:** Wei-Jian Li, Lu Zhang, Zi-Yao Chen, Wen-Zhu Shao, Liang Zhen

**Affiliations:** 1 College of Nuclear Equipment and Nuclear Engineering, Yantai University, Yantai 264005, China; 2 School of Materials Science and Engineering, Harbin Institute of Technology, Harbin 150001, China; 3 National Key Laboratory of Precision Hot Processing of Metals, Harbin Institute of Technology, Harbin 150001, China

**Keywords:** Cu matrix composite, CdMoO_4_, in situ synthesis, static contact resistance

## Abstract

Particle-reinforced Cu-based electrical contact materials prepared by traditional powder metallurgical methods suffer the same critical problem, where the agglomeration of the addition phases in the Cu matrix significantly deteriorates the performance of the composites and restricts their application. In this work, CdMoO_4_/Cu matrix composites were fabricated by an in situ method and followed by a powder metallurgical process. Firstly, CdMoO_4_/particles formed a nucleus and grew up based on the surfaces of Cu particles, realizing the controllable in situ synthesis of mixed powders with homogeneously dispersed CdMoO_4_ nanoparticles via a one-step reaction. Secondly, the bulk CdMoO_4_/Cu composites were fabricated by pressing and sintering and then densified by hot-extrusion and cold rolling processes. The microstructures and properties of the extruded and rolled specimens were characterized, respectively. The results indicated that the rolled CdMoO_4_/Cu composite exhibited excellent comprehensive properties of electrical conductivity and mechanical properties for electrical contact materials. Moreover, the effects of the contact force on the static contact resistance of the extruded and rolled composites were evaluated in the closed state of the contact materials. It was found that the rolled CdMoO_4_/Cu contact materials possessed a stable electrical contact characteristic with low and steady contact resistance. This work designed ternary CdMoO_4_ particles to reinforce Cu-based composites with well-balanced performances by an in situ synthesis method and this strategy can be extended to the design of ternary oxide/metal composites utilized as electrical contact materials.

## 1. Introduction

Copper is an attractive candidate to supersede precious metals utilized as electrical contact materials due to low cost and known superior electrical and thermal properties [1,2,3]. To satisfy the design requirements of electrical contact materials, numerous ceramic particles, such as Al_2_O_3_ [4], TiO_2_ [5], and ZrO_2_ [6], are generally introduced in the copper matrix to improve mechanical strength, wear, and arc resistance. However, the issue of inhomogeneous distribution of ceramic particles in the Cu matrix inevitably occurs in traditional preparation technologies, which significantly deteriorates the mechanical properties of Cu-based composites and limits their applications. To address this issue, an in situ method has aroused enormous interest and emerged as an effective route to synthesize dispersion-strengthened Cu-based composites. So far, extensive efforts have been dedicated to prepare TiO_2_/Cu [7], TiB_2_/Cu [8,9], and Al_2_O_3_/Cu [10] composites by an in situ method. The results indicated that the structures of the ceramic particle-reinforced Cu-based composites obtained by an in situ method were significantly improved with homogeneously distributed ceramic particles, and especially, these composites were proved to exhibit well-balanced electrical conductivity and mechanical properties.

Although the microstructures and properties of these oxide-reinforced Cu composites have been improved, the strong interfacial bonding of phase interfaces was still a major challenge due to the completely different characteristics of the constitutes. When subjected to the impacts from the frictional force, the cyclic loading, and the impact force, on one hand, the weak bonding between ceramic particles and the Cu matrix could easily induce crack initiation and propagation, resulting in serious failure of these traditional composites [11,12,13]. On the other hand, the weak interfacial adhesion provides a diffusion path for oxygen, leading to the internal oxidation of the Cu matrix at high temperatures. Additionally, under arc erosion, the reinforcement particles tend to agglomerate on the contact surface, which is ascribed to the weak adhesion between reinforcement and Cu [14,15,16,17]. The agglomeration phenomenon gives rise to the increasing of contact electrical resistivity and temperature, resulting in failure of contact material during long-time arc erosion. As a consequence, the design of durable Cu matrix electrical contact materials has aroused extreme interest.

In comparison to the binary ceramic phase, the ternary compound possesses potential application in electrical contact materials due to its strong adherence to the metal matrix. For example, a M*_n_*_+1_AX*_n_* phase such as Ti_3_AlC_2_ and Ti_3_SiC_2_ has been added in Cu matrices, which exhibited excellent arc-resistance performance [18,19,20,21]. A Zn_2_SnO_4_/Cu composite has been proved to decrease the mass loss under arc discharges, which was contributed to the strong ionic bonds across the phase interfaces [22]. In addition, Guo et al. [23] prepared Cu-based electrical contact materials reinforced by La_2_NiO_4_, which decreased the contact resistance to 21.6 mΩ from 29.5 mΩ for pure Cu, and simultaneously, the temperature rises of the designed La_2_NiO_4_/Cu degraded significantly due to the separation of oxides under arc erosion. However, so far, ternary compound-reinforced Cu-based composites applied in electrical contact material are quite limited. For example, the Ti_3_SiC_2_/Cu electrical contact materials were mainly utilized in the condition of vacuum [24]. For the La_2_NiO_4_/Cu composite, it claimed that the addition of La_2_NiO_4_ leaded to a serious mass loss in contrast to the pure Cu due to the self-cleaning functions [23]. As a result, the Ag/CdO and Cu/CdO contact materials remain used in aerospace industries, and even in domestic applications [25]. Thus, achieving highly reliable non-previous metal composites subjected to arc erosion in air is always full of challenge and significance.

Combining the composition design and structure regulation, we describe an in situ approach to directly produce the CdMoO_4_/Cu composite. Here, CdMoO_4_, exhibiting a combination characteristic of CdO as an arc-extinguishing agent and MoO_3_ as flame retardant, is proposed and expected to tailor the properties of Cu-based composite. Importantly, the introduction of MoO_3_, as the constituent of ternary oxides, can remarkably decrease the content of CdO in the composite. Simultaneously, CdMoO_4_/Cu mixed powders were synthesized with a one-step process by an co-precipitation method, expecting to improve the dispersion degree of reinforcement phases and enhance their adherence to the metal matrix. Unlike a previous in situ method, in which the metal is easily to be oxidized in acid solution and need to be reduced by hydrogen, the neutral conditions for CdMoO_4_ could protect Cu powders from oxidizing in solution.

In this work, to solve the problems of agglomeration and poor dispersion of the addition particles in the composites, we designed an in situ method with a one-step reaction to synthesize CdMoO_4_/Cu mixed powders, by which the CdMoO_4_ could homogeneously nucleate and grow on the surface of Cu particle to enhance the adhesion of phase interface. After pressing and sintering, hot-extrusion and cold rolling processes were employed to improve the properties, including density, electrical conductivity, and mechanical properties of 2 wt.% CdMoO_4_/Cu composites, and especially, realize the industrial production for the Cu-based electrical contact materials. To estimate the electric contact characteristic of the designed electrical contact materials, the static contact resistances were measured and the effects of the mechanism of the contact force on the static contact resistance of CdMoO_4_/Cu were investigated. The purpose of this work is providing a simple and effective strategy for Cu-based composites with homogeneously distributed CdMoO_4_ nanoparticles, and the strategy can be extended to other ternary oxide reinforced Cu-based composites to enlarge their applications.

## 2. Experimental

### 2.1. Fabrication of CdMoO_4_/Cu Mixed Powders and Composites

**Figure 1:**
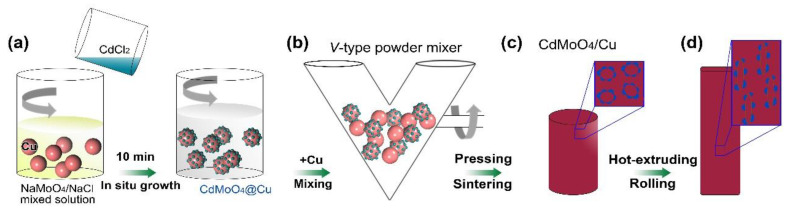
Schematic diagram of the fabrication of (**a**) CdMoO_4_/Cu mixed powders by an in situ method and (**b**–**d**) CdMoO_4_/Cu composites by powder metallurgy.

CdMoO_4_/Cu mixed powders were fabricated as follows (Figure 1a): 0.03 mol of Na_2_MoO_4_·2H_2_O and 0.06 mol of NaCl were added in 150 mL deionized water, and followed by adding 200 g electrolytic Cu powders (20~40 μm). The mixture was stirred continuously to form a suspension. Then, 0.03 mol CdCl_2_·2.5H_2_O were dissolved into 150 mL deionized water to get a clear solution. After that, the CdCl_2_ solution were mixed with the above suspension under continually stirring for 10 min. Finally, the powder particles in solution were filtrated and repeatedly washed. Subsequently, the 2 wt.%CdMoO_4_/Cu composites were prepared by powder metallurgy (Figure 1b–d), where 200 g electrolytic Cu powders were added in the dried CdMoO_4_/Cu powders. Here, such a content of CdMoO_4_ was promised to ensure a connected backbone structure of the Cu matrix, which, in view of the percolation theory [26,27], could provide transport paths for electric current. Excess CdMoO_4_ would completely encase the Cu particles and deduce the electrical and thermal conductivity by decreasing the percolation backbone density of Cu. After mixing (see reference [2]), the mixed powders were compacted under 250 MPa, and then green compact with a diameter of 80 mm was sintered at 910 °C in Argon atmosphere for 45 min. Then, the columnar specimen was processed into plate (6 × 50 mm^2^) by hot extruding at 800 °C. Afterwards, the rod material was cold rolled to a belt shape CdMoO_4_/Cu composite with a thickness of 2 mm, and then annealed at 500 °C for 30 min.

### 2.2. Characterizations

Five specimens with sizes of 2 × 10 × 10 mm^3^ were cut from the composites and used for the measurement of the real density and hardness. The relative density of the composites calculated by dividing the theoretical density into the real density, and the real density was measured based on Archimedes principle. The hardness measurement was carried on a Vickers hardness tester (HV-1000A, Hua Yin Test Instrument Co., Ltd., Yantai, China), and ten positions were chosen for each sample and the hardness was achieved by averaging the testing values. Five specimens in the sizes of 2 × 2 × 60 mm^3^ were cut from the plates along the extrusion and rolling directions, respectively and were used to measure the electrical conductivity by the four-probe method (Keithley 2420, Tektronix Inc., Beaverton, OR, USA) which was expressed in %IACS (International Annealed Copper Standard). Three samples for tensile testing with a gauge length of 18 mm and a cross-section of 1.5 × 6 mm^2^ were cut from the plates along the extrusion and rolling directions, respectively. Tensile tests were conducted at a strain rate of 5.6 × 10^−4^ s^−1^ using a universal testing machine (Instron-5569R, Boston, MA, USA) at room temperature, and the tensile test results were achieved by averaging the testing values. The static contact resistance was measured by mean of low-voltage alternating current contactor [2] installed with four CdMoO_4_/Cu specimens as movable and stationary contact materials, respectively. The adjustment of the gap distance between the contacts can realize the change of the contact force. Under a certain contact force, the static contact resistance can be achieved by the device of the simulation system with a four-probe method.

X-ray diffraction (D/Max 2500 system, Rigaku, Japan) was used to analyzed the phases and structure of CdMoO_4_/Cu powders. The scanning electron microscope (SEM) observation of the morphologies of the mixed powders and the fracture of the CdMoO_4_/Cu composites was implemented on FEI Quanta 200F microscope (Waltham, MA, USA). Here, the sizes of CdMoO_4_ particles and the grain were measured in Photoshop according the ruler. The structure of the composites was characterized with metallographic microscope (Axiovert 40 MAT, Zeiss, Oberkochen, Germany).

## 3. Results and Discussion

### 3.1. Characterization of the CdMoO_4_/Cu Mixed Powders and Composites

**Figure 2:**
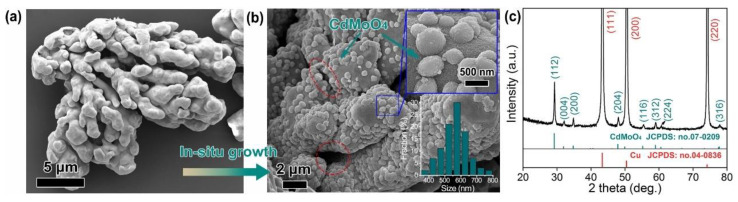
SEM images of (**a**) raw Cu powders and (**b**) as-synthesized CdMoO_4_/Cu mixed powders. (**c**) XRD pattern of the CdMoO_4_/Cu mixed powders. The inset in (**b**) is the high-magnification SEM image of CdMoO_4_/Cu mixed powders and corresponding size distribution diagrams for CdMoO_4_ particles.

The morphologies of the raw Cu powders and as-prepared CdMoO_4_/Cu powders, together with the XRD pattern of the obtained product, are shown in Figure 2. The dendritic morphology of the raw Cu in Figure 2a can provide large surface area for the growth of CdMoO_4_. After the in situ reaction, CdMoO_4_ particles with the sizes of 550~650 nm distributed uniformly on the surface of Cu powders (Figure 2b), especially between the branches of the Cu powders, as shown in the regions of red circles. The result shows that the in situ method can effectively solve the agglomeration of the CdMoO_4_ particles to ensure a uniform structure of the composite. High-magnification SEM image in Figure 2b shows that the CdMoO_4_ particles synthesized by an in situ method were hemispherical morphology, indicating strong adhesion to the Cu powder. Simultaneously, it can be observed that there exist gaps among the CdMoO_4_ particles without completely encasing the Cu powder as expected, which is beneficial to guarantee the electrical conductivity of the designed composites by forming continuous metallic passage. Additionally, Figure 2c shows that all diffraction peaks of the as-synthesized powders correspond to these of CdMoO_4_ (JCPDS no. 07-0209) and Cu (JCPDS no. 04-0836) without any impurities.

**Figure 3:**
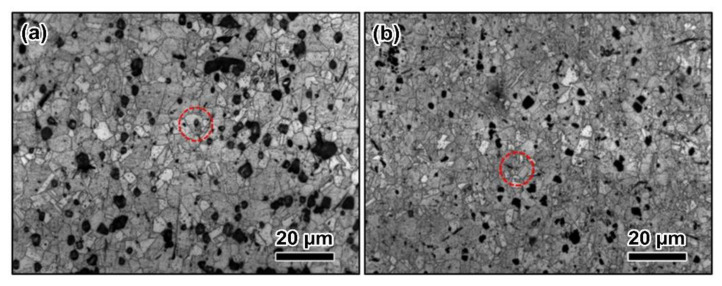
Optical images of the CdMoO_4_/Cu composites by powder metallurgy: (**a**) the hot-extrusion state and (**b**) the rolling state. CdMoO_4_ particles are indicated by red dashed circles.

Figure 3 shows the optical microstructures of CdMoO_4_/Cu composites, which were etched by FeCl_3_ solution. In Figure 3a, the grain size of extruded composite is approximately 10 μm. It can be detected that the CdMoO_4_ particles were distributed at the grain boundaries, while some of them are embedded in the grain interior, as shown in the reign of red dashed circles. It needs to point that the black region in Figure 3 indicates the peeling of the CdMoO_4_ particles from the composite surface after polishing. After rolled (Figure 3b), CdMoO_4_ were distributed homogeneously, including the grain interior and grain boundary. The CdMoO_4_ particles embedded in the grain interior are expected to improve the strength of the composite by hindering the movement of dislocations. Notably, the grain of the CdMoO_4_/Cu composites was obviously refined to approximately 5 μm by a rolling process, and the grain refinement can also contribute to the improvement of the strength of the CdMoO_4_/Cu composite. Moreover, the porosity, as a defect in the composites prepared via a powder metallurgical method, is one of the most important physical performance indexes, and excessive porosities could degrade the mechanical property and arc-resistance properties for the composites acting as electrical contact material. The relative densities (see Table 1) of the prepared composites were measured to be 99.0 ± 0.4% and 98.7 ± 0.6% for extruded and rolled specimens, respectively, which were significantly higher than those of reported ceramic/Cu [28,29] and [30,31] composites with the relative densities listed in Table 1. It reveals that the preparation technology involved in this work can effectively improve the density and decrease the porosity of the composites, which is the fundamental assurance of high reliability for electrical contact materials in practical applications.

### 3.2. Electrical Properties

The electrical conductivity of the extruded CdMoO_4_/Cu composite was measured to be 93.2 ± 1.1%IACS; however, the rolling technique cause a slight decrease on the electrical conductivity to a value of 89.1 ± 1.5%IACS. This highly relies on the changes of grain sizes discussed above, where electron scattering is slightly enhanced by the increasing grain boundary [35,36]. Nevertheless, the measured electrical conductivity is still higher than that of TiO_2_/Cu [32], Al_2_O_3_/Cu [33], Y_2_O_3_/Cu [34], and La_2_NiO_4_/Cu [23] electrical contact materials with values in the range of 34.7~85%IACS. The results reveal that the continuous metallic matrix in CdMoO_4_/Cu composites provides a pathway for electrical conduction, which fulfills the performance demands of contact materials.

### 3.3. Mechanical Properties

The hardness of the extruded and rolled specimens was tested and is listed in Table 1. In comparison of the rolled specimen, the extruded CdMoO_4_/Cu composite presents a relative lower hardness of 83.6 ± 3.4 HV0.1, from which it can be speculated that welding could easily occur between movable and stationary contact materials and thus, two contacts fail to disconnect under arc erosion. However, when the composite was further processed with cold rolling, the hardness of the composite was obviously increased 103.5 ± 1.8 HV0.1, which is ascribed to the CdMoO_4_ embedded in the grain interior and the grain refinement caused by cold rolling. Additionally, the measured results with small error bar can reflect a uniform structure of the rolled CdMoO_4_/Cu composite.

**Figure 4:**
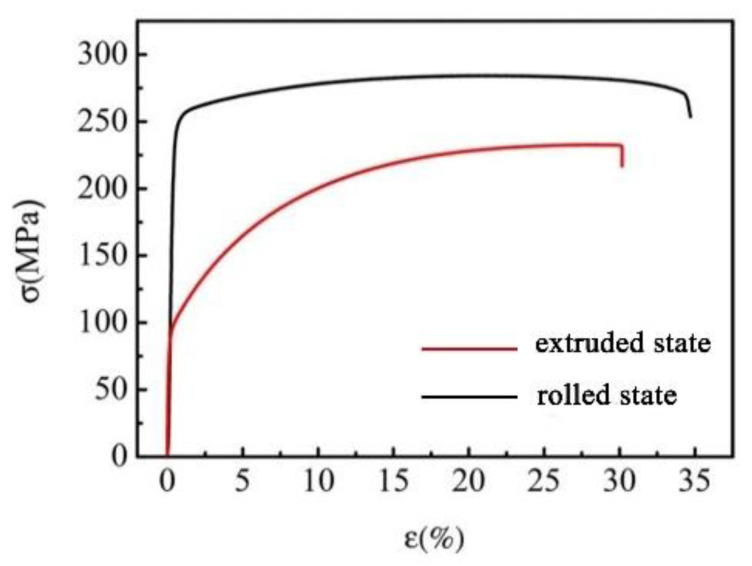
Strain-stress curves of extruded and rolled CdMoO_4_/Cu composites.

Figure 4 depicts the strain-stress curves of the extruded and rolled CdMoO_4_/Cu composites and the performance metrics are listed in Table 2. It reveals that the yield strength (245.9 ± 6.2 MPa) of the rolled composite is 145% higher than that of extruded composite (100 ± 3.7 MPa). Compared with the tensile strength of extruded composite (232.7 ± 5.7 MPa), the tensile strength of the rolled composite increases slightly to 261.8 ± 9.8 MPa. Except for the strength that decides the wear resistance and impact resistance during arc erosion, suitable ductility can ensure the factual contact area between the movable and stationary contact materials. The elongation of the extruded specimen is found to be 29.5 ± 0.9%, close to the values from previous studies (32–35.7%) [2,7,37]. It can be deduced that the in situ synthesized CdMoO_4_/Cu composites possess the ability of coordinate deformation, which is ascribed to the good interfacial coherent between CdMoO_4_ and the Cu matrix. Note that, unlike the previous studies where the mechanical properties were improved except for the ductility, the elongation of the CdMoO_4_/Cu composite increases from 29.5 ± 0.9 to 33.9 ± 0.8% after a rolling process, accompanying with the increasing of yield strength and tensile strength. This highly depends on the refined grains by a rolling process, which is consistent with the observation of the microstructure of the rolled composite. This testing was repeated at least three times, and same phenomena were achieved for the extruded and rolled CdMoO_4_/Cu specimens. The results indicate that the mechanical performance, especially for the ductility, of the composites was remarkably improved by the CdMoO_4_ particles by in situ fabrication.

**Figure 5:**
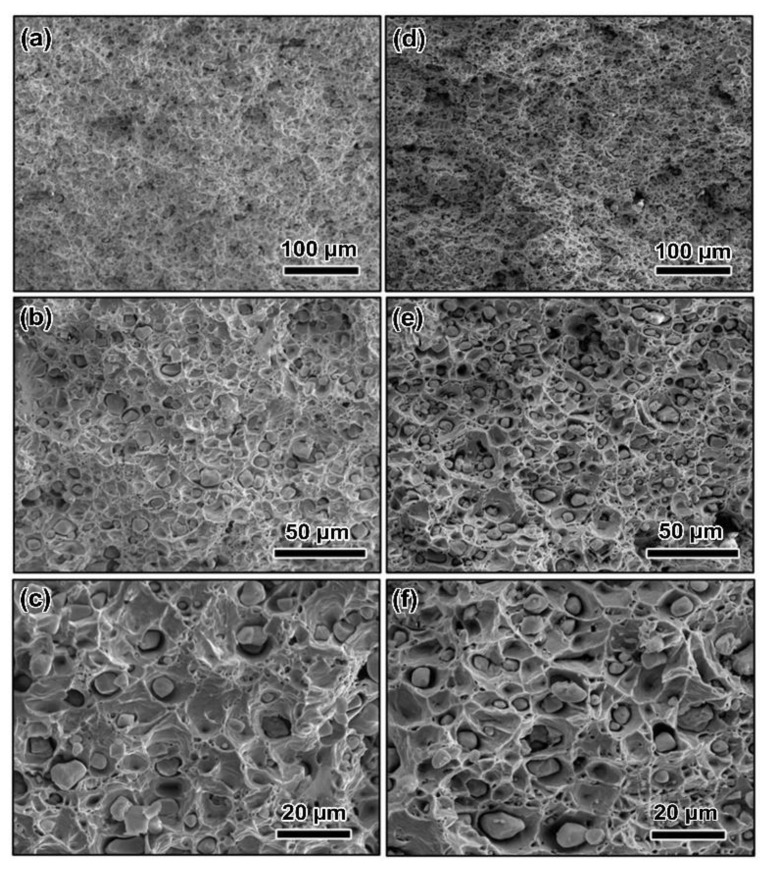
SEM images of the fracture of the (**a**,**c**,**e**) extruded CdMoO_4_/Cu composite and (**b**,**d**,**f**) the rolled CdMoO_4_/Cu composite.

The fracture morphologies of the CdMoO_4_/Cu composites are shown in Figure 5. The fracture behavior of the extruded composite reveals ductile properties with the evidence of large dimple-like fracture structure (Figure 5a,c,e). With regard to the rolled composite, smaller and deeper fracture dimples were generated, signifying that the rolling process increases the ductility of CdMoO_4_/Cu composites. These results are well consistent with those of the testing resulting of the mechanical performances of the CdMoO_4_/Cu composites.

### 3.4. Static Contact Resistance

**Figure 6:**
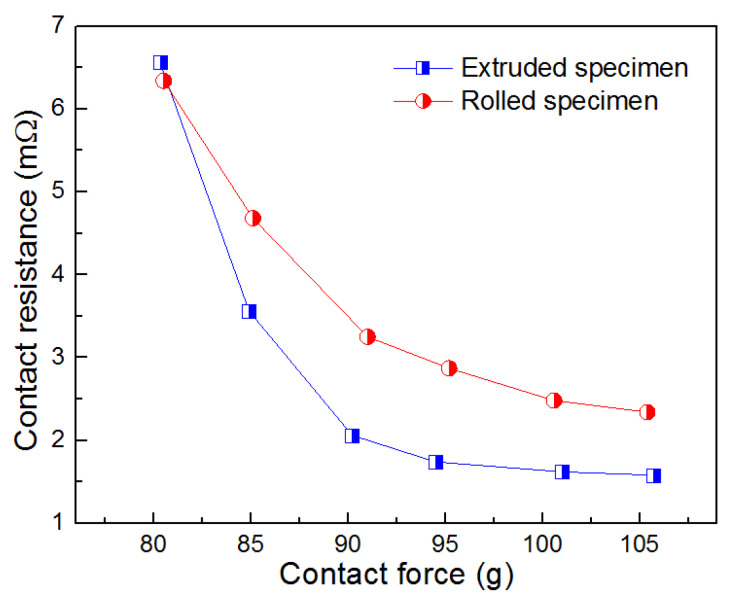
Changes of static contact resistance of CdMoO_4_/Cu composites with the contact force.

Static contact resistance is an important evaluation index that significantly impacts on the performance stability of contact materials under the connection state, and Figure 6 gives the static contact resistance of CdMoO_4_/Cu composites as a function of the contact force. As demonstrated in this figure, similar features can be observed for the extruded and rolled CdMoO_4_/Cu composites. Under the condition of the lower contact force (80 g), the two CdMoO_4_/Cu composites exhibited high contact resistance (6.5 mΩ). With increasing of the contact force, the static contact resistance of the composites decreased sharply until the contact force reached to 90 g, and then, the contact resistance decreased slowly. Finally, the contact resistances maintained stability in spite of the increasing of the contact force between the two contact materials. Note that, the electrical contact characteristic of rolled CdMoO_4_/Cu specimen enter the steady state rapidly under the contact force of 95 g, resulting in a lower contact resistance of 1.6 mΩ, in comparison with that (2.5 mΩ) of the extruded specimen (100 g). It indicated that the CdMoO_4_/Cu composite after rolling exhibited a more stable electrical contact characteristic, which is ascribed to excellent deformability for enlargement of the contact area. Additionally, the static contact resistance (1.6 mΩ) of the rolled CdMoO_4_/Cu composites is close to the contact resistance (1.55 mΩ) Cu contact materials as reported [38], and especially, the contact resistance of the prepared CdMoO_4_/Cu composites is lower than that of commercial Ag/CdO12 (approximately 5 mΩ) [39] and Ag/12SnO_2_ (approximately 1.6 mΩ) [40] electrical contact materials.

**Figure 7:**
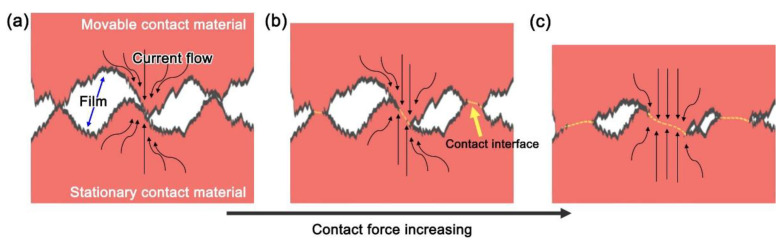
Schematic diagram of formation of contact resistance with increasing of the contact force. (**a**) Initial contact between the two contact materials, (**b**) Breakage of the film caused by increasing contact force, (**c**) Plastic deformation of the contact zone.

Figure 7 illustrate the schematic demonstration of the variation of the contact resistance with the contact force, which can be generally divided into three stages according to the dominant factors. First, under a low contact force (Figure 7a), the movable and stationary contact materials contact with their sharp protuberances due to the surface roughness. In this case, the current contract, resulting in an increasing of the flow path of the current, and thus, additional resistance, namely shrinkage resistance, is generated at the contact sites. Simultaneously, the surface films containing oxidation film, impurity, and chemical pollutants increase the contact resistance in the form of membrane resistance. At the second stage with an increased contact force (Figure 7b), these surface films were crushed by the contact force, decreasing the membrane resistance. Additionally, the contact area between the movable and stationary contact materials is enlarged and the shrinkage resistance is reduced. When further increasing the contact force (Figure 7c), sharp protuberances become flat, and the occurrence of work hardening leads to saturation point of the contact area [38], resulting in a stably contact resistance.

## 4. Conclusions

CdMoO_4_/Cu composites were prepared by an in situ method and followed by a powder metallurgical method. The effects of processing technologies on the structures and properties of the CdMoO_4_/Cu composites were investigated. The conclusions are as follows.

(1) CdMoO_4_/Cu mixed powders were successfully synthesized in a one-step reaction by an in situ method. The hemispherical CdMoO_4_ particles with the sizes of 500 nm grew uniformly on the Cu powders, realizing the controllable synthesis for ternary CdMoO_4_/Cu mixed powders with homogeneously dispersed reinforced phases. The proposed preparation method was determined to be a suitable technology for the particle-reinforced Cu-based composites.
(2) The CdMoO_4_/Cu composites were fabricated by a powder metallurgy method. In comparison with the extrude state of composite, the rolling process refined the grain from 10 to 5 µm and the CdMoO_4_ particles embedded in the grain interior.
(3) The rolled CdMoO_4_/Cu composite was verified to possess excellent comprehensive performances of relative density (98.7 ± 0.6%), electrical conductivity (89.1 ± 1.5%), hardness (103.5 ± 1.8 HV0.1), yield strength (245.9 ± 6.2 MPa), tensile strength (261.8 ± 9.8 MPa), and elongation (33.9 ± 0.9%).
(4) The variation tendencies of contact resistance of extruded and rolled CdMoO_4_/Cu composites with the contact force were evaluated. Small contact area and surface film caused a large contact resistance (approximately 6.5 mΩ) under a low contact force. A large contact force was applied to break the film of the contact surface, sharply decreasing the contact resistances. When hardening occurred and the contact area reached a saturation state, the contact resistances maintained stability.
(5) In comparison with the extruded specimen, the rolled CdMoO_4_/Cu composite with lower contact resistance (1.6 mΩ) exhibited a more stable electrical contact characteristic, which is ascribed to excellent deformability for enlargement of the contact area. The designed CdMoO_4_/Cu composite can be expected to use as electrical contact materials with a low and stable contact characteristic.

## Figures and Tables

**Table 1:** Relative density and electrical conductivity of CdMoO_4_-reinforced Cu-based composites.

	Relative Density (%)	Electrical Conductivity (%IACS)	Reference
Extruded specimen	99.0 ± 0.4	93.2 ± 1.1	This work
Rolled specimen	98.7 ± 0.6	89.1 ± 1.5	This work
3~9 wt.% ZrO_2_/Cu	90.2~94.5	——	[28]
Ti_3_SiC_2_/Cu	92.52~95	——	[29]
10~12 wt.% Ti_3_AlC_2_/Ag	92.4~92.6	——	[30]
Ag/Ni10	91.8~98.6	——	[31]
2.5 wt.% TiO_2_/Cu	——	78	[32]
2.75 wt.%Al_2_O_3_/Cu	——	85	[33]
2.5 wt.% Y_2_O_3_/Cu	——	37.8	[34]
5 wt.%La_2_NiO_4_/Cu	——	85	[23]

**Table 2:** Mechanical properties of CdMoO_4_-reinforced Cu-based composites.

	Hardness (HV0.1)	Yield Strength (MPa)	Tensile Strength (MPa)	Elongation (%)	Reference
Extruded specimen	83.6 ± 3.4	100.0 ± 3.7	232.7 ± 5.7	29.5 ± 0.9	This work
Rolled specimen	103.5 ± 1.8	245.9 ± 6.2	261.8 ± 9.8	33.9 ± 0.8	This work
2 wt.% SnO_2_/Cu	92.1	279.8	363.5	28.4	[2]
2 wt.% Zn_2_SnO_4_/Cu	103.8	289.4	369.2	35.7	[2]
0.82 wt.% TiO_2_/Cu	117.8 ± 6	290	——	32	[7]
0.2~0.8 wt.% CNTs/CuTi	88.22~100.86	152~192	266~352	15.1~28.2	[37]

## Data Availability

The data presented in this study are available on request from the corresponding author.
